# Magnitude and associated factors of cytopenias among antiretroviral therapy naïve *Human Immunodeficiency Virus* infected adults in Dessie, Northeast Ethiopia

**DOI:** 10.1371/journal.pone.0211708

**Published:** 2019-02-13

**Authors:** Zemenu Tamir, Abdurahaman Seid, Haftay Haileslassie

**Affiliations:** 1 Department of Medical Laboratory Sciences, College of Health Sciences, Addis Ababa University, Addis Ababa, Ethiopia; 2 Department of Medical Laboratory Sciences, College of Medicine and Health Sciences, Wollo University, Dessie, Ethiopia; 3 Department of Medical Laboratory Sciences, College of Health Sciences, Mekelle University, Mekelle, Ethiopia; Central University of Tamil Nadu, INDIA

## Abstract

**Background:**

Hematologic abnormalities involving peripheral blood cell cytopenias are strong predictors of morbidity, mortality and poor antiretroviral therapy (ART) outcomes of HIV infected individuals. However, limited studies are conducted in resource-limited settings of sub-Saharan Africa that have addressed the magnitude and associated factors of cytopenias. This study aimed to investigate the magnitude and associated factors of cytopenias among ART naïve HIV infected adult Ethiopians.

**Materials and methods:**

A cross-sectional study was conducted among ART naïve HIV infected individuals attending at ART unit of Dessie Referral Hospital between November 01, 2015 and April 30, 2016. A total of 402 adults were included using consecutive sampling. Socio-demographic, clinical and laboratory data of patients were collected. The data were entered to Epi Info version 3.4.3 and analyzed using SPSS version 20 software (SPSS INC, Chicago, IL, USA). Factors associated with cytopenias were analyzed first using bivariate and then multivariate logistic regression models. An odds ratio with 95% confidence interval was used to measure the strength of association. For all statistical significant tests, the cut-off value was set at P<0.05.

**Results:**

In this study, the overall magnitude of any cytopenia, anemia, leucopenia and thrombocytopenia were 63.4%, 43.5%, 24.4% and 18.7%, respectively. In multivariate logistic regression analysis, severe immunosuppression and WHO clinical stage IV HIV disease were significantly associated with increased prevalence of cytopenias. In addition, older age and younger age showed significant association with increased prevalence of anemia and leucopenia, respectively.

**Conclusion:**

Frequent occurrence of cytopenias was independently associated with severe immunosuppression and WHO clinical stage IV HIV disease. Further longitudinal multicenter studies are recommended to bolster the findings of this study in order to suggest the need of routine assessment and management of hematological abnormalities for optimal choice of initial antiretroviral agents and prevention of further morbidities.

## Introduction

Human Immunodeficiency Virus (HIV) infection and Acquired Immunodeficiency Syndrome (AIDS) encompass a clinical spectrum of diseases in which cytopenias of all blood cell lines are encountered especially in ART naïve patients [1, 2]. Cytopenias including anemia, leucopenia and thrombocytopenia are among the most common non immunological complications of HIV infection [3, 4]. The pathophysiological basis for the development of cytopenias may include impaired hematopoiesis, immune-mediated cytopenias, and coagulopathies especially in the advanced stage of the disease [5, 6].

Prevalence of cytopenias has been shown to vary geographically, with the highest prevalence of anemia and neutropenia among treatment-naive patients with AIDS from Africa than among those from Asia or America [7, 8]. The incidence and severity of cytopenias also correlate with the progression of the disease from the asymptomatic HIV carrier state to the advanced state of the disease [2, 9]. A systematic review of the literature documented the prevalence or incidence of anemia in HIV-infected patients ranges from 1.3% to 95% depending on the stage of HIV/AIDS disease and definition of anemia, making it more common than thrombocytopenia or leucopenia in HIV/AIDS patients [10, 11]. Thrombocytopenia, which is also a frequent complication of HIV infection with a different geographic distribution from anemia and neutropenia[7], is found in 3–40% of patients and could occur at any stage of HIV infection [2, 12–14]. Leucopenia is one of the hematological abnormalities that occur in the course of HIV disease progression. Its prevalence is widely variable ranging from 10–50% [3, 15, 16].

Although hematological parameters are neither part of the criteria for initiating therapy nor used by the World Health Organization (WHO) for staging HIV disease, peripheral blood cell abnormalities are important prognostic tools for poor treatment outcome and morbidity in HIV infection and AIDS [17–19]. Especially in developing countries, where access to the health institutions is low, people will visit these institutions after the disease progressed to a severe state accompanied by cytopenias; hematological parameters have a paramount significance. Few studies have been conducted in resource-limited settings of sub-Saharan Africa that have addressed the magnitude of peripheral blood cell cytopenias which may have a considerable impact on the patient’s wellbeing and treatment; and associated factors for cytopenias that might help to develop strategies to reduce its incidence.

Therefore, this study was conducted to assess the magnitude and associated factors of cytopenias among ART naïve HIV infected adults attending at the ART unit of Dessie Referral Hospital, Dessie, Northeast Ethiopia.

## Materials and methods

### Study design, setting and participants

This cross-sectional study was conducted at Dessie Referral Hospital, Dessie, Northeast Ethiopia. Dessie Referral Hospital is found in Dessie city, which is about 401 kilometers away from Addis Ababa, the capital city of Ethiopia. The hospital serves as a referral center for the surrounding zones and provides comprehensive health care services including HIV/AIDS interventions such as free diagnosis, treatment and monitoring.

This study was conducted from November 1, 2015 to April 30, 2016 and involved a total of 402 consecutively selected HIV infected individuals. The study participants were comprised of ART naïve HIV-infected individuals who had been followed up or newly enrolled at the adult ART unit of Dessie Referral Hospital during the study period. Individuals who had been previously confirmed of having chronic renal failure and liver disease prior to HIV infection; those who underwent radiation therapy and/or mylosuppresive chemotherapy in the previous 45 days; and HIV infected pregnant mothers were excluded from the study due to the fact that these may induce or exacerbate cytopenias by themselves.

### Data collection procedure

Patients’ socio-demographic data was collected using a structured pretested questionnaire in a face to face interview. Clinical data was collected by reviewing medical records of study participants. Patients were weighed to the nearest 0.1 kilogram in light indoor clothing and bare feet or with stockings. Height was measured using a stadiometer after participants stood in erect posture without shoes, and the results were recorded to the nearest 0.5 centimeters. Body mass index (BMI) was calculated as the ratio of weight in kilograms to the square of height in meters. The aforementioned data was collected by ART trained nurses. During data collection, the principal investigator supervised the data collection and examined the tools for accuracy and completeness.

### Laboratory measurements and definitions of hematological abnormalities

Hematological and immunological data of study participants was collected after analysis of blood by trained medical laboratory technologists. About 4ml of venous blood was collected from each study participant using EDTA coated test tubes. The hematological parameters: total white cell count(WBC), absolute red blood cell count (RBC), Platelet count, total lymphocyte count and hemoglobin concentration (Hgb) values, were determined using the hematology analyzer Cell-Dyn 1800 (Abbott Laboratories Diagnostics Division, USA) and CD4+ T cells were assayed using the BD FACSCOUNT system (Becton Dickenson and Company, California, USA). All laboratory procedures were performed under strict adherence of the standard operational procedures to maintain the quality of data. The low, medium and high quality control materials were run along with patient samples to assure the performance of the instruments.

Anemia was defined using the WHO criteria after adjustment for the altitude of the study area based on the WHO guideline [20]. Accordingly, anemia was defined as Hgb concentration <13 g/dl for males and < 12 g/dl for females. We classified anemia as mild (11–12.9g/dl for males and 11–11.9g/dl for females), moderate (8–10.9 g/dl) and sever (<8 g/dl)[20]. Leucopenia was defined as total white blood cell (WBC) count < 4 × 10^9^/L and thrombocytopenia was defined as total platelet count < 1.5× 10^11^ cells/L) [3]. Any cytopenia, bicytopenia and pancytopenia were defined as presence of at least one form of cytopenias (anemia, leucopenia or thrombocytopenia), two forms and all forms of cytopenias, respectively.

### Statistical analysis

The data were entered into Epi Info version 3.4.3 software and exported to Statistical package for Social Sciences (SPSS) version 20.0 statistical software for analysis. Normally distributed and non-normally distributed continuous variables were presented as mean ± standard deviation (SD) and median (inter quartile range (IQR)), respectively.

Bivariate logistic regression model was used to assess the relationship between HIV-associated cytopenias and independent variables. A multivariate logistic regression model was used to identify the independent determining factors of cytopenias among variables which had P-valve ≤0.3 in the bivariate analysis. Odds ratio (OR) with 95% confidence interval (CI) was used to measure the strength of statistical association. P value < 0.05 was used to indicate statistical significance.

### Ethical consideration

The study was approved after the protocol was reviewed by the research and ethical committee of Wollo University, College of Medicine and Health Sciences. Permission to conduct the study was obtained from Dessie Referral Hospital. Written informed consent was obtained from each study participant and all patient identifiers were removed and only code numbers were used to keep confidentiality of data throughout the study.

## Results

### Demographic and clinical characteristics of participants

In this study, a total of 402 ART naïve HIV infected adults who had been followed up or newly enrolled at the ART unit of Dessie Referral Hospital from November 01, 2015-April 30, 2016 were included. The majority of the study participants were females (55.7%). The mean ± SD age of study participants was 36.2 ± 9.5 years, ranging from 18 to 65 years, and 89.3% of them were below 50 years old. The mean ± SD BMI was 19.4±2.9 and 61.4% of them were normal weight. About 58% of study participants had a positive history of opportunistic infections (OI) and majority (52.8%) of them were at advanced clinical stage of HIV/AIDS disease (WHO clinical stage III or IV) [Table 1].

**Table 1:** Demographic and clinical characteristics of ART naïve HIV infected adults attending at Dessie Referral Hospital from November 1, 2015-April 30, 2016, Dessie, Northeast Ethiopia. (N = 402).

Characteristics	Value
**Age(years), mean ± SD**	36.2±9.5
**Age group, n(%)**	
18–29 years	101(25.1)
30–39 years	165(41.1)
40–49 years	93(23.1)
≥50 years	43(10.7)
**Sex, n(%)**	
Male	178(44.3)
Female	224(55.7)
**BMI, median(IQR)**	19.4±2.9
**BMI category, n(%)**	
Underweight	138(34.3)
Normal	247(61.4)
Overweight	17(4.3)
**Opportunistic Infection, n(%)**	
No	169(42)
Yes	233(58)
**WHO clinical stage, n(%)**	
Stage I or II	190(47.3)
Stage III or IV	212(52.7)
**CD4**^**+**^ **T-cell count(cells/mm^3^), median(IQR)**	183(116–329)
**CD4**^**+**^ **count (cells/mm^3^), n(%)**	
<200	244(60.7)
200–249	80(19.9)
350–499	32(8.0)
≥500	46(11.4)
**Hemoglobin(g/dl), mean ±SD**	12.3±2.92
**WBC count(cells/mm^3^), median(IQR)**	5.1x10^3^ (4.0–6.5x10^3^)
**Platelet count(cells/mm^3^), median(IQR)**	2.44x10^5^ (1.77–3.09x10^5^)

The median (IQR) CD4^+^ T-cell count was 183(116–329) cells/mm^3^ and 88.6% of participants had CD4^+^ T-cell count < 500 cells/mm^3^. The median (IQR) of leukocyte and thrombocyte count was 5.1× 10^3^ (4.0–6.5) cells/mm^3^ and 2.44×10^5^ (1.77–3.09) cells/mm^3^, respectively. The mean ± SD Hgb concentration was 12.3 ± 2.92g/dl [Table 1].

### Prevalence and associated factors of cytopenias

In this study, the overall prevalence of any cytopenia and pancytopenia was 63.4% and 4.2%, respectively. The anemia—leucopenia combination was the most frequent bicytopenia, 13.9%, followed by anemia-thrombocytopenia which was 8.5% [Table 2].

**Table 2:** Magnitude of cytopenias among ART naïve HIV infected adults attending at Dessie Referral Hospital from November 1, 2015-April 30, 2016, Dessie, Northeast Ethiopia (N = 402).

Peripheral Blood Abnormality	Yes,n(%)	No,n(%)
**Any Cytopenia**^**a**^	255(63.4)	157(36.6)
**Pancytopenia**	17(4.2)	385(95.8)
**Bicytopenia**		
Anemia and Leucopenia	56(13.9)	346(86.2)
Anemia and thrommbocytopenia	34(8.5)	368(91.5)
Thrombocytopenia and leucopenia	20(5)	382(95)
**Anemia**	175(43.5)	277(56.5)
**Anemia Severity**^**b**^		
Mild	69(17.2)	33(82.8)
Moderate	70(17.4)	332(82.6)
Severe	36(8.9)	366(91.1)
**Leucopenia**	98(24.4)	304(75.6)
**Thombocytopenia**	75(18.7)	327(81.3)

^a^ presence of at least one cytopenias anemia severity among the total anemia cases

^b^ prevalence of anemia severity was computed from the total participants

In the bivariate logistic regression analysis, the presence of any cytopenia was significantly associated with history of opportunistic infections (P = 0.001), being underweight in BMI (P = 0.037) and being at advanced or severely immunosuppressed situation (P<0.01). After adjustment for confounding variables using multivariate logistic regression analysis, advanced immunosuppression and severe immunosuppression were identified as independent predictors for presence of at least one form of cytopenia. Study participants with CD4+ T-cell count of 200–349 cells/mm^3^ and <200 cells/mm^3^ were seven and fifteen times more likely to have at least one form of cytopenias than those who had CD4 count ≥500cells/mm^3^, (OR = 7, P = 0.01) (OR = 15.2, P<0.001), respectively [Table 3].

**Table 3:** Association of demographic and clinical characteristics with magnitude of total cytopenia among ART naïve HIV infected adults attending at Dessie Referral Hospital from November 1, 2015-April 30, 2016, Dessie, Northeast Ethiopia (N = 402).

Characteristics	Any Cytopenia		Bivariate logistic regression analysis		Multivariate logistic regression analysis	
	Yes (n(%))	No (n(%))	COR^c^[95%CI]	P-Value	AOR^@^[95%CI]	P-value
**Gender**						
Female	140(62.5)	84(37.5)	1			
Male	115(64.5)	63(35.4)	1.1[0.73,1.65]	0.66		
**Categorized Age**						
18–29 years	64(63.4)	37(36.6)	1.09[0.61,1.95]	0.76		
30–39 years	107(64.8)	58(35.2)	1.16[0.69,1.97]	0.57		
40–49 years	57(61.3)	36(38.7)	1			
**≥50 years**	27(62.8)	16(37.2)	1.06[0.5,2.25]	0.87		
**WHO Stage**						
Stage I&II	115(61.5)	72(38.5)	1		1	
Stage III & IV	140(65.1)	75(34.9)	1.17[0.78,1.75]	0.45	1.35[0.77,2.37]	0.3
**Categorized BMI**						
Overweight	8(47.1)	9(52.9)	0.56[0.22,1.57]	0.27	1.2[0.37,3.85]	0.77
Normal	149(60.3)	98(39.7)	1		1	
Under weight	98(71)	40(29)	1.6[1.03,2.52]	0.037	1.5[0.9,2.46]	0.12
**Opportunistic Infection**						
Absent	90(53.3)	79(46.7)	1		1	
Present	165(70.8)	68(29.2)	2.13[1.41,3.2]	0.001	1.3[0.57,2.22]	0.36
**Categorized CD4 count (cells/mm^3^)**						
>500	8(17.4)	38(82.6)	1		1	
350–499	11(35.5)	20(64.5)	2.6[0.9,7.5]	0.076	2.27[0.74,7.03]	0.15
200–349	47(57.3)	35(42.7)	6.4[2.65,15.4]	0.001	7.0[2.68,18.2]	0.01
<200	189(77.8)	54(22.2)	16.6[7.3,37.7]	<0.001	15.2[6.3,36.8]	<0.01

^c^ COR—Crude Odds Ratio,

^@^AOR—-Adjusted Odds Ratio.

The overall magnitude of anemia in this study was 43.5%; of which 17.2%, 17.4% and 8.9% were mild, moderate and severe anemia types, respectively [Table 2]. Anemia prevalence was 44.9% among males and 42.4% among females, and gender had no significant influence on the overall prevalence of anemia (*P* = 0.611). The overall prevalence of anemia ranges from 37.6%-53.5% among different age group categories of participants. Anemia did not show significant difference among different age groups (P>0.05) [Table 4].

**Table 4:** Association of demographic and clinical characteristics with anemia among ART naïve HIV infected adults attending at Dessie Referral Hospital from November 1, 2015-April 30, 2016, Dessie, Northeast Ethiopia (N = 402).

Characteristics	Anemia		Bivariate logistic regression analysis		Multivariate logistic regression analysis	
	Yes (n(%))	No (n(%))	COR^c^[95%CI]	P-Value	AOR^@^[95%CI]	P-value
**Gender**						
Female	95(42.4)	129(57.6)	1			
Male	80(44.9)	98(55.1)	1.11[0.74,1.64]	0.611		
**Categorized Age**						
40–49 years	35(37.6)	58(62.4)	1		1	
18–29 years	43(42.6)	58(57.4)	1.23[0.69,2.18]	0.43	1.1[0.58,2.14]	0.75
30–39 years	74(44.8)	91(55.2)	1.35[0.8,2.27]	0.26	1.44[0.8,2.6]	0.22
≥50 years	23(53.3)	20(46.5)	1.91[0.92,3.96]	0.084	2.43[1.06,5.56]	0.032
**WHO Stage**						
Stage I	24(32.9)	49(67.1)	1		1	
Stage II	52(45.9)	62(55.4)	1.71[0.93,3.16]	0.085	2.2[0.94,5.23]	0.069
Stage III	68(42.2)	93(57.8)	1.49[0.84,2.67]	0.176	1.52[0.72,3.2]	0.27
Stage IV	31(57.4)	23(42.6)	2.75[1.33,5.69]	0.006	2.29[1.13,4.64]	0.022
**Categorized BMI**						
Overweight	3(17.6)	14(82.4)	1		1	
Normal	102(41.3)	145(58.7)	3.28[0.92,11.2]	0.062	1.45[0.36,5.92]	0.603
Under weight	70(50.7)	68(49.3)	4.8[1.32,17.46]	0.017	2.1[0.49,8.79]	0.32
**Opportunistic Infection**						
Absent	52(30.8)	117(69.2)	1		1	
Present	123(52.8)	110(47.2)	2.52[1.66,3.81]	0.001	1.54[0.8,2.37]	0.25
**Categorized CD4 count (cells/mm^3^)**						
>500	5(10.9)	41(89.1)	1		1	
350–499	6(19.4)	25(80.6)	1.97[0.54,2.3]	0.32	1.13[0.29,4.34]	0.86
200–349	20(24.4)	62(75.6)	2.9[0.98,8.58]	0.072	1.74[0.56,5.4]	0.334
<200	144(59.3)	99(40.7)	11.9[4.53,31.3]	<0.001	7.7[2.76,21.48]	0.001
**Leucopenia**						
No	119(38.8)	188(61.2)	1		1	
Yes	56(58.9)	39(41.1)	2.27[1.42,3.63]	0.001	1.56[0.93,2.64]	0.06
**Thrombocytopenia**						
No	141(43.1)	186(56.9)	1			
Yes	34(45.3)	41(54.7)	1.09[0.66,1.81]	0.727		

^c^ COR—Crude Odds Ratio,

^@^AOR—-Adjusted Odds Ratio.

In this study, anemia prevalence increases with decreasing CD4^+^ T-cell count. Its overall prevalence was 59.3%, 24.4%, 19.4% and 10.9% among patients with CD4^+^ T-cell count of <200 cells/mm^3^, 200–349 cells/mm^3^, 350–499 cells/mm^3^ and ≥500 cells/mm^3^, respectively. A significant association was noted between anemia and CD4 T-cell count less than 200 cells/mm^3^ (P = 0.001). Anemia also had significant association with WHO clinical stage IV HIV disease (P = 0.006), positive history of opportunistic infections (P<0.001) and lower body weight (P = 0.017). It also showed significant association with leucopenia (P<0.001) but not with thrombocytopenia (P = 0.727).

After adjustment for confounding variables using multivariate logistic regression modeling, anemia was independently predicted by age ≥50 years, WHO clinical stage IV HIV disease and CD4 count < 200 cells/mm^3^. Older study participants (≥50 years) were 2.43 times more likely to be anemic than individuals with age range of 40–49 years, (OR = 2.43, P = 0.032). Similarly, ART naïve HIV infected individuals at WHO clinical stage IV HIV disease were 2.29 times more likely to be anemic than WHO clinical stage I participants, (OR = 2.29, P = 0.022). In addition, severely immunosuppressed ART naïve HIV infected individuals (CD4 counts < 200 cells/mm^3^) were 7.7 times more likely to be anemic than immunocompetent counterparts (CD4 count ≥500 cells/mm3), (OR = 7.7, P <0.001), as summarized in Table 4.

Leucopenia was present among 24.4% of study participants [Table 2]. The overall prevalence of leucopenia was 21.3% and 26.8% among males and females, respectively; and gender had no statistically significant influence in leucopenia prevalence (p = 0.208). Leucopenia prevalence showed a decreasing trend with increment of age, ranging from 11.6%-33.7% within different age categories. It showed significant association with lower age (18–29 years), P = 0.009. Leucopenia had also a decreasing trend with increasing CD4^+^ T-cell counts, which ranges from 30.5% -4.3% within the different CD4 categories. It has a significant association with CD4^+^ T-cell count of <200 cells/mm^3^ (P = 0.004) and from 200–349 cells/mm^3^ (P = 0.019). It also showed significant association with history of opportunistic infection (P = 0.017), WHO clinical stage IV HIV disease (P = 0.009) and anemia (P = 0.002). However, leucopenia did not show significant association with body mass index and thrombocytopenia (P>0.05) [Table 5].

**Table 5:** Association of demographic and clinical characteristics with Leucopenia among ART naïve HIV infected adults attending at Dessie Referral Hospital from November 1, 2015-April 30, 2016, Dessie, Northeast Ethiopia (N = 402).

Characteristics	Leucopenia		Bivariate logistic regression analysis		Multivariate logistic regression analysis	
	Yes (n(%))	No (n(%))	COR^c^[95%CI]	P-Value	AOR^@^[95%CI]	P-value
**Gender**						
Male	38(21.3)	140(78.7)	1		1	
Female	60(26.8)	164(73.2)	1.35[0.85,2.14]	0.208	1.31[0.78,2.2]	0.309
**Categorized Age**						
≥50 years	5(11.6)	38(88.4)	1		1	
40–49 years	16(17.2)	77(82.8)	1.58[0.54,4.63]	0.406	1.63[0.53,5.10]	0.93
30–39 years	43(26.1)	122(73.9)	0.27[0.99,7.25]	0.052	2.92[1.04,8.2]	0.034
18–29 years	34(33.7)	67(66.3)	3.86[1.39,10.7]	0.009	3.79[1.3,11.26]	0.015
**WHO Stage**						
Stage I	19(26)	55(74)	1		1	
Stage II	28(24.6)	88(75.4)	0.65[0.32,4.03]	0.15	0.54[0.99,5.67]	0.41
Stage III	31(19.3)	130(80.7)	0.27[1.21,3.87]	0.134	0.31[0.87,3.48]	0.113
Stage IV	20(37)	34(63)	2.47[1.25,4.85]	0.009	2.6[1.32,5.85]	0.001
**Categorized BMI**						
Overweight	2(11.8)	15(88.2)	1		1	
Normal	60(24.3)	187(75.7)	2.5[0.53,10.83]	0.26	1.01[0.18,6.51]	0.89
Under weight	36(26.1)	102(73.9)	2.65[0.58,12.15]	0.21	1.2[0.19,7.23]	0.77
**Opportunistic Infection**						
Absent	31(18.3)	138(81.7)	1		1	
Present	67(28.8)	166(71.2)	1.8[1.1,2.91]	0.017	1.4[0.77,2.55]	0.27
**Categorized CD4 count (cells/mm^3^)**						
>500	2(4.3)	44(95.7)	1		1	
350–499	4(12.9)	27(87.1)	6.67[0.71,62.8]	0097	6.7[0.67,67.6]	0.106
200–349	18(22)	64(78)	11.8[1.5,91.6]	0.019	15.2[1.94,119]	0.009
<200	74(30.5)	169(69.5)	19.3[2.6,142.8]	0.004	16.7[2.0,129.9]	0.001
**Anemia**						
No	42(18.5)	185(81.5)	1		1	
Yes	56(32)	119(68)	2.07[1.31,3.29]	0.002	1.56[0.92,2.68]	0.102
**Thrombocytopenia**						
No	78(23.9)	249(76.1)	1			
Yes	20(26.7)	55(73.5)	1.16[0.67,2.06]	0.61		

^c^ COR = Crude Odds Ratio,

^@^AOR = Adjusted Odds Ratio.

After adjustment using multivariate logistic regression analysis, younger age from 18–29 years, and 30–39 years, WHO clinical stage IV HIV diseases, CD4 counts from 200–349 cells/mm^3^ and less than 200cells/mm^3^ were identified as independent predictors of leucopenia. Study participants within 18–29 years and 30–39 years range were about 3.79 and 2.92 times more likely to be leucopenic than participants ≥50 years old, (OR = 3.79, P = 0.015; OR = 2.92, P = 0.034) respectively. Study participants with WHO clinical stage IV HIV disease were 2.6 times more likely to be leucopenic than those with WHO clinical stage I disease, (OR = 2.6, P = 0.01). Similarly patients with CD+4 T-cell count from 200–349 cells/mm^3^ and less than 200 cells/mm^3^ were 15.2 and 16.7 times more likely to be leucopenic than immunocompetent participants (≥500cells/mm3), OR = 15.2, P = 0.009; OR = 16.7, P <0.001, respectively [Table 5].

Thrombocytopenia was present among 18.7% of study participants [Table 2]. The overall prevalence of thrombocytopenia was 20.2% and 17.4% among males and females, respectively; and gender had no statistically significant influence in thrombocytopenia (p = 0.555). Similarly thrombocytopenia did not show significant association within different age group categories, BMI categories, history of opportunistic infections, anemia and leucopenia (P>0.05). However, thrombocytopenia increased with advancement of HIV/AIDS disease, ranging from 15.1%-35.2% among WHO clinical stage I to IV, respectively; and it had significant association with stage IV HIV disease (P = 0.01). Similarly thrombocytopenia increased with decreasing CD^+^4 T-cell count; being 6.5% - 22.5% within different CD^+^4 T-cell count categories and showed significant association with CD4 count <200 cells/mm^3^ [P = 0.02] [Table 6].

**Table 6:** Association of demographic and clinical characteristics with Thrombocytopenia among ART naïve HIV infected adults attending at Dessie Referral Hospital from November 1, 2015-April 30, 2016, Dessie, Northeast Ethiopia (N = 402).

Characteristics	Thrombocytopenia		Bivariate logistic regression analysis		Multivariate logistic regression analysis	
	Yes (n(%))	No (n(%))	COR^c^[95%CI]	P-Value	AOR^@^[95%CI]	P-value
**Gender**						
Female	39(17.4)	185(82.6)	1			
Male	36(20.2)	142(79.8)	1.2[0.727.1.99]	0.47		
**Categorized Age**						
18–29 years	17(16.8)	84(83.2)	1		1	
30–39 years	25(15.2)	140(84.8)	0.88[0.45,1.73]	0.72	0.82[0.33,2.01]	0.66
40–49 years	24(25.8)	69(74.2)	1.72[0.85,3.45]	0.13	1.49[0.62,3.6]	0.37
≥50years	9(20.9)	34(79.1)	11.3[0.53,3.2]	0.56	1.4[0.55,3.55]	0.48
**WHO Stage**						
Stage I	11(15.1)	62(84.9)	1		1	
Stage II	18(15.8)	96(84.2)	1.06[0.47,2.39]	0.894	1.55[0.62,2.34]	0.346
Stage III	27(16.8)	134(83.2)	1.14[0.53,2.44]	0.744	1.46[0.6,3.75]	0.43
Stage IV	19(35.2)	35(64.8)	3.06[1.31,7.16]	0.01	5.36[1.98,14.4]	0.001
**Categorized BMI**						
Under weight	23(16.7)	115(83.3)	1			
Normal	48(19.3)	199(80.7)	1.21[0.69,2.08]	0.502		
Over weight	4(23.5)	13(76.5)	1.54[0.46,5.14]	0.482		
**Opportunistic Infection**						
Absent	26(15.4)	143(84.6)	1		1	
Present	49(21)	184(79.0)	1.46[0.87,2.47]	0.15	1.13[0.59,2.15]	0.61
**Categorized CD4 count (cells/mm^3^)**						
>500	3(6.5)	43(93.5)	1		1	
350–499	2(6.5)	29(93.5)	0.99[0.16,6.28]	0.99	0.67[0.1,4.53]	0.68
200–349	16(19.5)	66(80.5))	3.47[0.96,12.6]	0.06	3.4[0.87,13.3]	0.077
<200	54(22.2)	189(77.8)	4.1[1.2,13.7]	0.02	3.64[1.03,12.9]	0.045
**Anemia**						
No	41(18.1)	186(81.9)	1			
Yes	34(19.4)	141(80.6)	1.094[0.66,1.81]	0.727		
**Leucopenia**						
No	55(18.1)	249(81.9)	1			
Yes	20(20.4)	78(79.6)	1.16[0.66,2.06]	0.61		

^c^ COR—Crude Odds Ratio,

^@^AOR—-Adjusted Odds Ratio.

In multivariate logistic regression analysis, WHO clinical stage IV HIV diseases and severe immunosuppression (<200 cells/mm^3^) were identified as independent predictors for thrombocytopenia. Patients at WHO clinical stage IV HIV diseases and those who were at severe immunosuppression were about 5.36 and 3.64 times more likely to be thrombocytopenic than WHO clinical stage I and immunocompetent individuals, (OR = 3.64, P = 0.045; OR = 3.64, P = 0.045), respectively.

## Discussion

This study was intended to assess the magnitude and associated risk factors of peripheral cytopenias among ART naïve HIV infected adults in a resource-scarce setting. Accordingly, it showed that at least one form of cytopenia was present among 63.4% of the study participants. Anemia was present among 43.5% of study participants followed by leucopenia (24.4%) and thrombocytopenia (18.7%). Prior study reports on HIV associated cytopenias among ART naïve individuals were less consistent with our findings. Studies conducted in Uganda [21], Nigeria [3] and South Korea [22] reported 65%, 59.8% and 11.2% of the study participants had at least one form of blood cytopenias, respectively. The difference might be due to variation in methodology of the research, socio-demographic and clinical characteristics of the study population and definition of cytopenias used in each study. For instance the South Korean study, which was conducted by analyzing patients data records retrospectively, had used strict exclusion criteria such as patients with opportunistic infections or other signs of infectious illness, with any malignancy or who had received chemotherapeutic agents within six months prior to enrollment or patients with medication history for agents which can induce cytopenias within two weeks before enrolment; it also defined anemia if Hgb concentration was less than 10g/dl [22].

Being the most frequent cytopenia in almost all previous studies and an independent risk factor for HIV/AIDS disease progression, the magnitude of anemia was variable based on the study settings and the definition of anemia. Our finding of anemia which was identified as the most frequent among cytopenias was in agreement with other findings which reported from 42.3%-43.8% [2, 21, 23]. On the other hand, the finding of this study was lower than studies which revealed between 49.5% and 65.5% [3, 12, 24, 25], and higher than other studies which reported between 3% and 29.9% prevalence [13, 18, 22, 26].

In HIV/AIDS patients increased platelet destruction, either caused by the nonspecific deposition of circulating immune complexes on platelets or by the presence of specific anti-platelet antibodies, as well as direct infection of megakaryocytes by HIV resulting with ineffective platelet production was assumed to be the possible pathogenic features of thrombocytopenia [3,27,28]. Hence, chronic thrombocytopenia is relatively common hematologic complication of HIV disease. In the current study, prevalence of thrombocytopenia (18.7%) which is similar to studies in Nigeria [13] and Uganda [14]; was higher than reports from Northwest Ethiopia [2, 18, 28] and Uganda [21] but much lower than reports from Cameroon [29] and Turkey [30]. The variation in prevalence among different studies could be due to the difference in methodology as well as socio-demographic and clinical characteristics of the study population.

In HIV patients’ the cause of leucopenia could be reduced bone marrow activity from infiltrative conditions, increased apoptosis and HIV infection itself [25]. Leucopenia is, therefore, a common finding in ART naïve patients with a prevalence that ranges between 10% and 44% [31, 32] that may be associated with increased risk of severe bacterial infection and hospitalization [25]. The prevalence of leucopenia in this study was comparable with studies conducted in Uganda [21] and Tanzania [25]; higher than studies in Ethiopia [2, 18] and lower than studies in Nigeria [13] and Cameroon [29]. The observed difference in magnitude of different studies could be due to the study population demographic difference, sample size difference and leucopenia definition variability.

Magnitude of pancytopenia and bicytopenia in this study was nearly in agreement with other studies. A study conducted in Tanzania reported 5.1% prevalence of pancytopenia and as anemia-leucopenia combination was the most frequent bicytopenia [25]. On the other hand, our finding was in contrast with a worldwide study conducted in Asia, Africa and America which reported 0.3% pancytopenia; and 1.2% had both neutropenia and thrombocytopenia, 0.9% had both anemia and thrombocytopenia and 2.2% had both anemia and neutropenia [7]. The difference was due to the cut off value used to define cytopenias in the worldwide study (neutropenia if absolute neutrophil count ≤ 1.3 × 10^9^/l, anemia If Hgb ≤ 10 g/dl and thrombocytopenia if platelets ≤ 125 × 109/l) and population differences between the Americans, Europeans and other Africans from Ethiopians.

Cytopenias in ART naïve HIV infected individuals is multifactorial. HIV-associated hematologic abnormalities in these individuals may involve direct HIV infection of bone marrow progenitors, abnormal regulation of hematopoiesis and/or autoimmune phenomena [33, 34]. Previous studies conducted to investigate hematological abnormalities among treatment–naïve HIV patients indicated that the degree of cytopenias were directly related to advancement of HIV disease where the immune system is substantially debilitated, viral replication has not been controlled and opportunistic infections are rampant [21, 35, 36]. In this study, CD4 T-cell count from 200–349 cells/mm^3^ and <200 cells/mm^3^ were identified as significant independent predictors for presence of any cytopenias. This supports the fact that bone marrow suppression and production defects are caused by HIV and the severity of cytopenias is correlated with severity of the disease.

Different studies conducted in different parts of the world indicated that anemia is directly related to the degree of immunosuppression and severity of HIV Diseases [3, 13, 33]. In this study, old age (≥ 50 years), stage IV HIV disease and CD4^+^ T-cell count <200 cells/mm^3^ were identified as independent predictors of anemia. The finding was in agreement with a review of literatures by Patel reported increased prevalence of anemia as a function of age after the fifth decade of life in both men and women [37]. Similarly, a Chinese study reported increased prevalence of anemia with increasing age and decreasing CD4^+^ T-cell count; and had identified older age as one of the significantly associated factors with an increased risk of anemia [33].

Studies indicated that progression of HIV infection measured by decreasing CD4+ T- cell count, advanced WHO clinical stage of HIV disease and increased HIV-RNA were associated with increased risk of leucopenia [2, 3, 9, 15]; therefore, leucopenia is a bad prognostic indicator of HIV disease [36]. In agreement with this, our study showed significantly increased prevalence of leucopenia among study participants with WHO clinical stage IV HIV disease and lower CD4 count. In addition to this, although not supported by other studies, the current study showed increased prevalence of leucopenia with relatively younger age.

Isolated thrombocytopenia is frequently the first hematological manifestation of HIV infection [38]. However, studies showed that thrombocytopenia is more prevalent and severe among patients with more advanced HIV disease or lower CD4^+^ T cell count [13,25,39]. In agreement with these studies, the current study revealed that thrombocytopenia was present among 15.1% of WHO clinical stage I participants; and independently predicted by WHO clinical stage IV HIV disease and severe immunosuppression but not with manifestation of leucopenia or anemia. This affirms that isolated thrombocytopenia might occur early in the natural history of HIV infection otherwise in asymptomatic HIV sero-positive cases as well as in advanced HIV disease with a clinical evolution different from anemia and leucopenia which occur frequently in combination [38, 40].

## Conclusion

This study showed high prevalence of peripheral blood cell cytopenias among ART naïve individuals living with HIV/AIDS in resource-limited settings. Lower CD4^+^ T-cell count and WHO clinical stage IV HIV disease were identified as independent predictors for presence of anemia, leucopenia and thrombocytopenia. Moreover, although the findings of the current study indicated the necessity of emphasizing on diagnosis and management of hematological abnormalities among ART naïve HIV infected individuals in order to provide an optimal choice of initial antiretroviral agents and to prevent further morbidities, additional multicenter longitudinal research is recommended to generalize the related outcomes of this study.
